# Supplementary material for: Global Phylogeny and F Virulence Plasmid Carriage in Pandemic Escherichia coli ST1193
Source: Microbiol Spectr. 2022 Nov 21;10(6):e02554-22. doi: 10.1128/spectrum.02554-22 (PMC9769970; doi:10.1128/spectrum.02554-22)
Supplement: Supplemental file 3 — Legends of Tables S1 and S2. Download spectrum.02554-22-s0003.pdf, PDF file, 0.1 MB [file spectrum.02554-22-s0003.pdf]

Supplemental Table 1 Metadata and genotyping information for *Escherichia coli* ST1193 whole genome sequences.

Supplemental Table 2 Metadata and genotyping information of *Escherichia coli* isolates from animal agriculture on EnteroBase.
